# Promoting health and wellbeing through building the relationship with build and cultural heritage

**DOI:** 10.1192/j.eurpsy.2023.2100

**Published:** 2023-07-19

**Authors:** E. Abdelmoula, N. Bouayed Abdelmoula

**Affiliations:** 1 2M2RCA, ENAU Ecole Doctorale Sciences et Ingénierie Architecturales (ED-SIA), Tunis; 2 Genomics of Signalopathies at the service of Medicine, Medical University of Sfax, Sfax, Tunisia

## Abstract

**Introduction:**

For over two decades, much has been written about the role of heritage buildings and historic places on individual and societal well-being. Numerous manuscripts have been devoted to study the relationship between built and cultural heritage and well-being and to decrypt their mental and physical health benefits at the personal and societal levels. Other studies have focused on the diverse types of heritage experiences that enable these health benefits, ranging from perceptions, to the individual and societal engagements through exposure, protection and conservation of historic places and environment. Social benefits encompass enhancing of collective memories and identities, sharing experiences, social interaction, creative opportunities, etc.

**Objectives:**

The objective of this research is to explore the relationship between personal and societal health and well-being, and cultural heritage. The main objective is to disclose international strategies and programs for assessing and promoting heritage-led wellbeing benefits.

**Methods:**

We comprehensively reviewed the scientific literature using qualitative data-analysis methods to state, international frameworks for promoting heritage-led wellbeing benefits.

**Results:**

Our bibliographic review revealed that there has been rising recognition that the conservation of cultural heritage is not just about the preservation of materials, but rather about safeguarding and sharing heritage for the improvement of people’s lives. According to the WHO, culture and cultural heritage are recognized as fundamental determinants of what makes personal and social lives meaningful. Consequently, many countries such as the UK and Canada, have developed people centred approaches achieved by local authorities as a means of ensuring inclusivity and diversity, so that heritage is able to contribute to people’s wellbeing. Politically, few agendas promoting wellbeing and engagement with the historic environment included guidelines of community-oriented projects and management systems that benefit people with building the relationship between people and place, while demonstrating the public value of the historic environment.

**Conclusions:**

All professionals, at the architectural and urban level, as well as health and psychology level should have awareness about the concept of heritage-led wellbeing benefits and strategic views of the value of cultural heritage as process, as participation, as mechanism, as healing, as place and as environment to raise the planned positive effects by local authorities when promoting the perception of the role of cultural heritage in health and wellbeing.

**Disclosure of Interest:**

None Declared

